# Modulation of chloride homeostasis by inflammatory mediators in dorsal root ganglion neurons

**DOI:** 10.1186/1744-8069-4-32

**Published:** 2008-08-12

**Authors:** Katharina Funk, Anne Woitecki, Christina Franjic-Würtz, Thomas Gensch, Frank Möhrlen, Stephan Frings

**Affiliations:** 1 Department of Molecular Physiology, University of Heidelberg, Im Neuenheimer Feld 230, 69120 Heidelberg, Germany; 2 Institute for Neuroscience and Biophysics 1, Juelich Research Center, Leo-Brand-Strasse, 52425 Juelich, Germany

## Abstract

**Background:**

Chloride currents in peripheral nociceptive neurons have been implicated in the generation of afferent nociceptive signals, as Cl^- ^accumulation in sensory endings establishes the driving force for depolarizing, and even excitatory, Cl^- ^currents. The intracellular Cl^- ^concentration can, however, vary considerably between individual DRG neurons. This raises the question, whether the contribution of Cl^- ^currents to signal generation differs between individual afferent neurons, and whether the specific Cl^- ^levels in these neurons are subject to modulation. Based on the hypothesis that modulation of the peripheral Cl^- ^homeostasis is involved in the generation of inflammatory hyperalgesia, we examined the effects of inflammatory mediators on intracellular Cl^- ^concentrations and on the expression levels of Cl^- ^transporters in rat DRG neurons.

**Results:**

We developed an *in vitro *assay for testing how inflammatory mediators influence Cl^- ^concentration and the expression of Cl^- ^transporters. Intact DRGs were treated with 100 ng/ml NGF, 1.8 μM ATP, 0.9 μM bradykinin, and 1.4 μM PGE_2 _for 1–3 hours. Two-photon fluorescence lifetime imaging with the Cl^-^-sensitive dye MQAE revealed an increase of the intracellular Cl^- ^concentration within 2 hours of treatment. This effect coincided with enhanced phosphorylation of the Na^+^-K^+^-2Cl^- ^cotransporter NKCC1, suggesting that an increased activity of that transporter caused the early rise of intracellular Cl^- ^levels. Immunohistochemistry of NKCC1 and KCC2, the main neuronal Cl^- ^importer and exporter, respectively, exposed an inverse regulation by the inflammatory mediators. While the NKCC1 immunosignal increased, that of KCC2 declined after 3 hours of treatment. In contrast, the mRNA levels of the two transporters did not change markedly during this time. These data demonstrate a fundamental transition in Cl^- ^homeostasis toward a state of augmented Cl^- ^accumulation, which is induced by a 1–3 hour treatment with inflammatory mediators.

**Conclusion:**

Our findings indicate that inflammatory mediators impact on Cl^- ^homeostasis in DRG neurons. Inflammatory mediators raise intracellular Cl^- ^levels and, hence, the driving force for depolarizing Cl^- ^efflux. These findings corroborate current concepts for the role of Cl^- ^regulation in the generation of inflammatory hyperalgesia and allodynia. As the intracellular Cl^- ^concentration rises in DRG neurons, afferent signals can be boosted by excitatory Cl^- ^currents in the presynaptic terminals. Moreover, excitatory Cl^- ^currents in peripheral sensory endings may also contribute to the generation or modulation of afferent signals, especially in inflamed tissue.

## Background

The regulation of intracellular chloride, [Cl^-^]_i_, in DRG neurons has a distinct impact on the detection and transmission of the peripheral nociceptive signals. Measurements with isolated DRG neurons have shown that Cl^- ^is accumulated into the cytoplasm, supporting a Cl^- ^equilibrium potential, E_Cl_, near -40 mV [1-3]. The opening of Cl^- ^permeable channels, therefore, induces a depolarizing Cl^- ^efflux. Presynaptic GABA_A _receptors in the dorsal horn conduct Cl^- ^efflux and cause primary afferent depolarization (PAD) [4,5]. PAD mediates presynaptic inhibition of nociceptive afferents, but intense afferent stimulation can cause excitatory Cl^- ^efflux which contributes to dorsal root reflexes, hyperalgesia, and neurogenic inflammation [4-8]. In addition to these well-characterized spinal processes, excitatory Cl^- ^effects were also reported for the cutaneous endings of nociceptors [9,10]. Together, these observations raise the possibility that depolarizing Cl^- ^currents contribute to signal generation in the periphery as well as to synaptic transmission. Interestingly, [Cl^-^]_i _can increase under certain conditions, as was recently reported for nociceptors upon nerve damage and regeneration (*e.g*. from 31 to 68 mM [11]). A rising level of [Cl^-^]_i _is expected to amplify peripheral and presynaptic Cl^- ^efflux and its effect on sensory signal generation and transmission. As a consequence of these findings, the regulation of Cl^- ^transport proteins has become an important topic of nociceptor research [12]. Indeed, a number of recent studies has documented a link between pain behaviour and Cl^- ^accumulation in cutaneous and visceral nociceptors. Genetic ablation of the Na^+^-K^+^-2Cl^- ^cotransporter NKCC1 was associated with reduced Cl^- ^accumulation in DRG neurons and caused a reduced thermal nociceptive response [13] as well as attenuation of Aβ-mediated allodynia [14]. TRPV1-dependent, referred abdominal allodynia [15], itch and flare responses [16], as well as nocifensive behaviour in phase II of the formalin test [17], were all inhibited upon spinal or peripheral application of the NKCC1-inhibitor bumetanide. Similarly, dorsal root reflexes and neurogenic inflammation were attenuated by intrathecal bumetanide injection [18].

Cl^- ^regulation in neurons is mediated by cation-coupled Cl^-^-cotransporters (CCCs) [19-21]. NKCC1 (SLC12A2) is the main key player of active Cl^- ^uptake. In contrast, Cl^- ^extrusion in neurons is mediated by the K^+^-Cl^- ^cotransporter KCC2 (SLC12A5). For DRG neurons, the expression of NKCC1 is well documented [13,22], while the expression of KCC2 is controversial [23,24]. The intracellular Cl^- ^concentration in neurons is mainly determined by the functional expression of these CCCs. In addition, regulatory mechanisms that control the activity of Cl^- ^transporters include phosphorylation [25-27] and dimerization [28]. Importantly, small shifts in the balance between Cl^- ^import and Cl^- ^extrusion can cause changes of E_Cl _which substantially alter a neuron's response to the opening of Cl^- ^channels. A recent approach to model the GABAergic response of a spinal lamina 1 neuron revealed that a shift of E_Cl _as small as 10 mV to less negative values is sufficient to turn GABA-induced currents from inhibitory to excitatory [29]. Interestingly, a recent thermodynamic analysis of Cl^- ^transport in DRG neurons indicated that the regulation of NKCC1 activity by phosphorylation/dephosphorylation may account for substantial changes in E_Cl _[3]. Thus, Cl^- ^levels in DRG neurons are probably not constant, but they are subject to regulation. Processes that control the dynamics of Cl^- ^accumulation, consequently, play an important role in current concepts for the plasticity of the afferent pain pathway [3,5,8,12,30-35].

Here we examined the hypothesis that [Cl^-^]_i _is modulated by factors involved in inflammatory hyperalgesia. We studied the effects of inflammatory mediators on the intracellular Cl^- ^concentration as well as on the expression of NKCC1 and KCC2. We used an *in vitro *assay to monitor Cl^- ^concentrations in intact, freshly dissected DRGs by two-photon fluorescence lifetime imaging microscopy (2P-FLIM). We find that inflammatory mediators cause an increase of Cl^- ^levels in DRG neurons which correlates with enhanced NKCC1 expression and decreased KCC2 expression.

## Results

### *In vitro *assay for monitoring effects of inflammatorymediators

**Figure 1:**
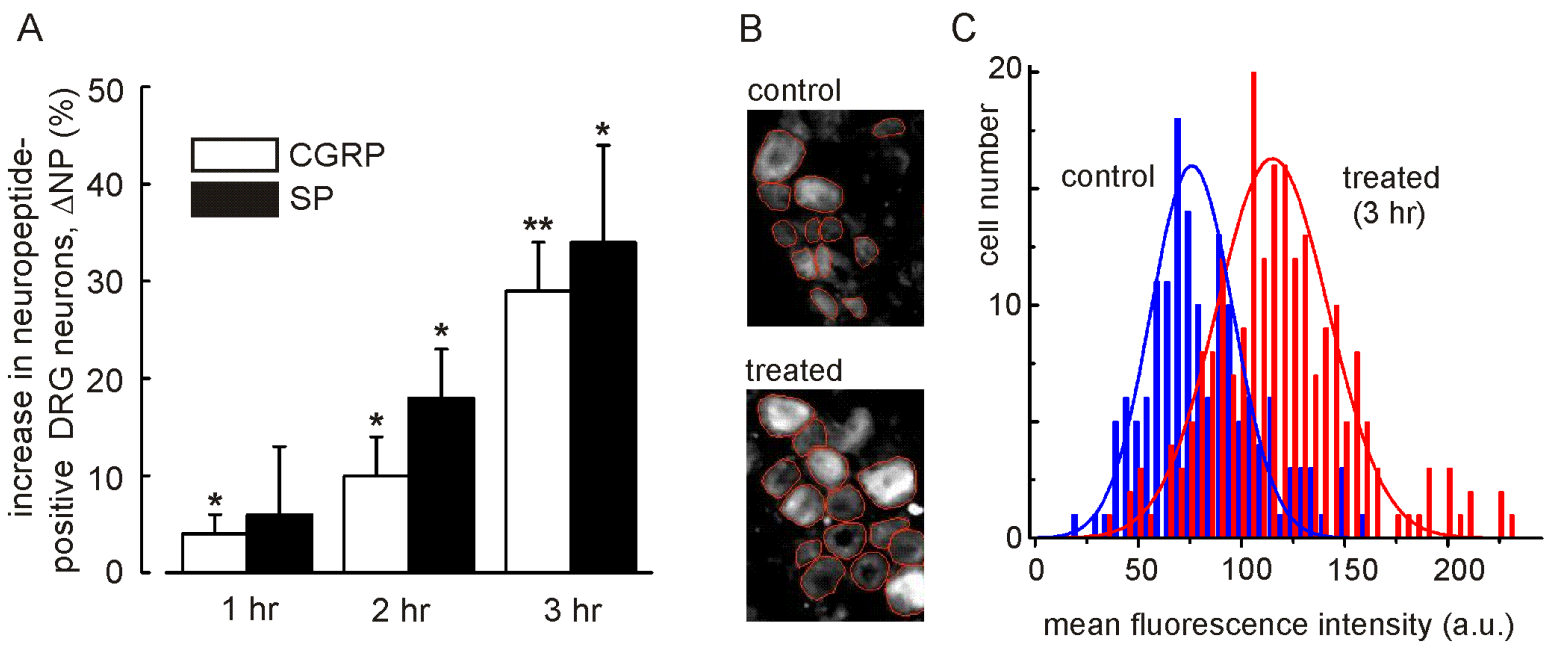
***In vitro *assay to study the effects of inflammatory mediators by immunohistochemistry**. (A) Increased expression of neuropeptides during treatment with inflammatory mediators. The immunfluorescence of calcitonin gene-related peptide (CGRP) and substance P (SP) was evaluated at the indicated times after start of the treatment. The relative number of neuropeptide-expressing neurons was determined in test DRGs and in contralateral control DRGs, and the percent change of positive cells, Δ NP = (NP_test_-NP_ctr_)/NP_ctr_·100 [%], is given for each time. Indicated significance levels are p ≤ 0.05 (*) or p ≤ 0.01 (**). (B) Cryosections of a control DRG and the treated contralateral test DRG stained with NKCC1 antibodies. The red circles illustrate the area from which the mean fluorescence intensity was obtained. Background subtraction removed most of the pericellular fluorescence as well as the fluorescence originating from the nucleus. All cell sections with intact perimeter were evaluated. (C) Distribution of fluorescence intensities recorded from 3 control DRGs *(blue) *and 3 contralateral test DRGs *(red) *illustrate a shift to higher intensities after a 3-hr treatment with inflammatory mediators. Solid lines are Gaussian fits to the data.

Our objective was to monitor changes of the intracellular Cl^- ^concentration as well as changes in expression of NKCC1 and KCC2 in the presence of inflammatory mediators. To minimize damage to the neurons and to avoid dedifferentiation effects that may alter Cl^- ^homeostasis in cell culture, we examined the neurons in rat DRGs with intact *dura mater*. The DRGs were excised and immediately used for the assay which was concluded within < 4 hr *post mortem*. The excised DRGs were incubated with a combination of inflammatory mediators (100 ng/ml NGF, 1.8 μM ATP, 0.9 μM bradykinin, 1.4 μM PGE_2_) for 1, 2, or 3 hr at 37°C, and then either subjected to 2P-FLIM analysis or fixed for immunohistochemistry. The vitality of DRG neurons during this procedure was tested in DRG tissue slices using propidium-iodide exclusion and resazurin-reduction assays (see Methods). Both tests confirmed a cell viability of > 95% within the 3-hr period of experimentation with and without the inflammatory mediators *(not shown)*. To find out whether the DRG neurons responded to the inflammatory mediators, we analyzed the immunosignals of the neuropeptides CGRP and substance P (SP) which are known to be upregulated during inflammation [*e.g*. [36-38]]. We compared the relative numbers of neuropeptide-expressing neurons in DRGs treated with inflammatory mediators to control DRGs from the same animal treated with exactly the same protocol, but without the inflammatory mediators. In 9 control DRGs (T7-T9) from 3 animals, we found on average 35% of the neurons expressing CGRP and 33% expressing SP. The 9 corresponding contralateral test DRGs were treated with inflammatory mediators for 1 hr (3 T7-DRGs from 3 rats), 2 hr (3 T8-DRGs), or 3 hr (3 T9-DRGs). We determined the relative number of neuropeptide-positive cells separately for each control DRG (NP_ctr_) and for its contralateral test DRG (NP_test_), and we quantified the percent change of neuropeptide-positive neurons according to Δ NP = (NP_test_-NP_ctr_)/NP_ctr_·100 [%]. Fig. 1A illustrates the time-dependent increase of neuropeptide-positive neurons in the treated ganglia. Δ NP (± SD; 3 animals) was 4 ± 2% (1 hr), 10 ± 4% (2 hr), and 29 ± 5% (3 hr) for CGRP, and 6 ± 7% (1 hr), 18 ± 5% (2 hr), and 34 ± 10% (3 hr) for SP. In total, 5329 control cells and 4596 test cells were counted for the CGRP statistics, and 3178 control cells and 3467 treated cells for SP. These data show that the DRG neurons in our *in vitro *assay respond to the inflammatory mediators with enhanced synthesis of neuropeptides, a behaviour which is indicative of an inflammatory response *in vivo*. To monitor changes of [Cl^-^]_i _between control and treated DRGs we examined the Cl^-^-dependent fluorescence of MQAE by 2P-FLIM immediately after the test time of 1, 2, or 3 hours, as described below.

For immunohistochemical studies, we prepared cryosections of the control DRGs and of test DRGs and stained the cells with antibodies directed against NKCC1 or KCC2. We then evaluated the mean fluorescence intensity of each immunostained DRG neuron as described in Methods. Fig. 1B shows fluorescence images to illustrate the evaluation procedure. Following background subtraction, the mean fluorescence intensity of each intact and uniformly stained cell in a section was measured using identical imaging parameters. This analysis resulted in a pair of histograms (control and test) of the cell fluorescence intensities for each treatment time (Fig. 1C; example for 3 hr). The mean fluorescence intensities in control DRGs (FI_ctr_) and corresponding test DRGs (FI_test_) were determined by Gaussian fits to the histograms and were used to quantify the percent change of immune fluorescence ΔFI = (FI_test_-FI_ctr_)/FI_ctr_·100 [%] upon treatment with inflammatory mediators.

### Inflammatory mediators cause enhanced Cl^- ^accumulation

**Figure 2:**
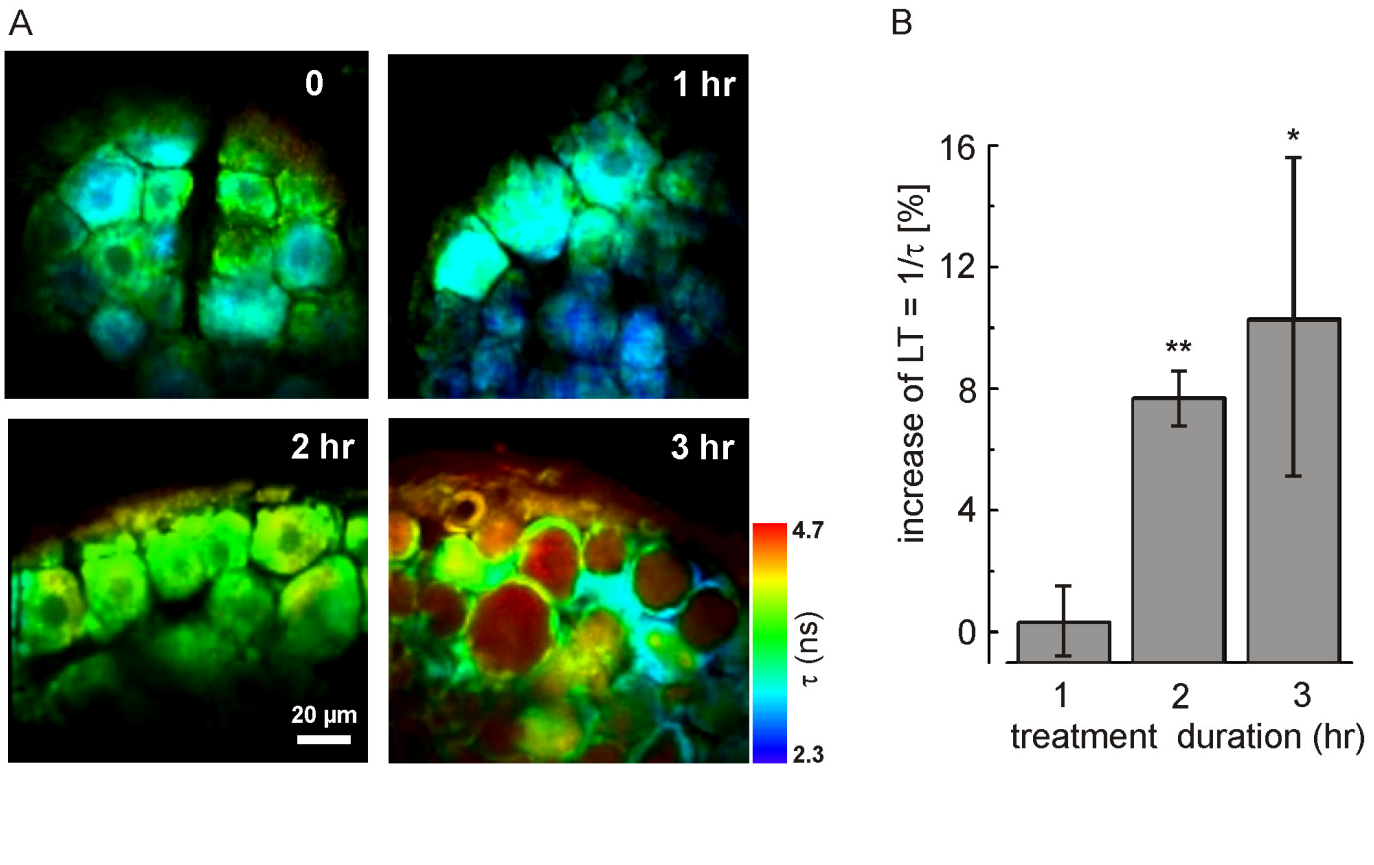
(A) Two-photon fluorescence lifetime imaging microscopy (2P-FLIM) of intact DRGs loaded with the chloride indicator dye MQAE. The colour code indicates the fluorescence lifetime (τ) which is inversely proportional to the Cl^- ^concentration. Warm colours represent high intracellular Cl^- ^concentration (small τ values). (B) Quantitative evaluation of 2P-FLIM data show a highly significant increase of the inverse lifetime (LT = 1/τ) two hours after the start of treament with inflammatory mediators. Mean LT values were determined in control DRGs (LT_ctr_) and in contralateral test DRGs (LT_test_), and the percent increase of LT is depicted as ΔLT = (LT_test_-LT_ctr_)/LT_ctr_·100 [%] for each treatment time. Indicated significance levels are p ≤ 0.05 (*) or p ≤ 0.01 (**).

To look for changes of the intracellular Cl^- ^concentration during exposure to inflammatory mediators, we loaded the DRG neurons with the Cl^-^-sensitive fluorescent dye MQAE. MQAE fluorescence is quenched by Cl^- ^and can therefore be used to monitor time-dependent changes of the intracellular Cl^- ^level. To obtain an experimental parameter which depends only on changes of intracellular Cl^-^, we analyzed the fluorescence lifetime of MQAE [39-41]. Recordings were taken from intact DRGs (T7 – T9) by focusing the light of a two-photon microscope through the *dura mater *into cell layers 20 – 100 μm deep in the DRG. The recorded fluorescence lifetime was colour-coded for the microscopic images such that warmer colours represent higher Cl^- ^concentrations (Fig. 2A). We did not attempt to calibrate the 2P-FLIM signals in terms of absolute Cl^- ^concentrations (see Discussion). We used the inverse of the fluorescence lifetime τ (LT = 1/τ) as a parameter that is proportional to the Cl^- ^concentration, according to 1/τ = 1/τ_0 _+ (K_SV _[Cl^-^]_i_)/τ_0_, where τ_0 _is the value of τ in Cl^-^-free solution, and K_SV _is the Stern-Volmer constant. Fig. 2A illustrates that the Cl^- ^levels rise in most DRG neurons 2 hr after addition of the inflammatory mediators, and show a further increase after another hour. For the 2P-FLIM analysis, 18 DRGs (9 treated, 9 control) from 6 animals (in total, 365 control cells and 361 test cells) were evaluated. For each treatment time, the mean inverse lifetime, LT, was determined for all neurons in a control DRG (LT_ctr_) and the contralateral test DRG (LT_test_). Corresponding to our evaluation of immunostains, we determined the percent increase of LT as ΔLT = (LT_test_-LT_ctr_)/LT_ctr_·100 [%]. The data in Fig. 2B demonstrate that Cl^- ^increased significantly within 2 hr of treatment with inflammatory mediators. ΔLT (± SD; 3 animals) was -0.3 ± 1.2% (1 hr), 7.7 ± 0.9% (2 hr), and 10.3 ± 5.3% (3 hr). Moreover, the 2P-FLIM measurements revealed that virtually all DRG neurons visible in the images increase their intracellular Cl^- ^concentrations in response to the inflammatory stimulus. Thus, DRG neurons respond to inflammatory mediators more or less uniformly by boosting the driving force for depolarizing Cl^- ^efflux.

### Changes in expression of Cl^- ^transporters correlate with Cl^-^accumulation

**Figure 3:**
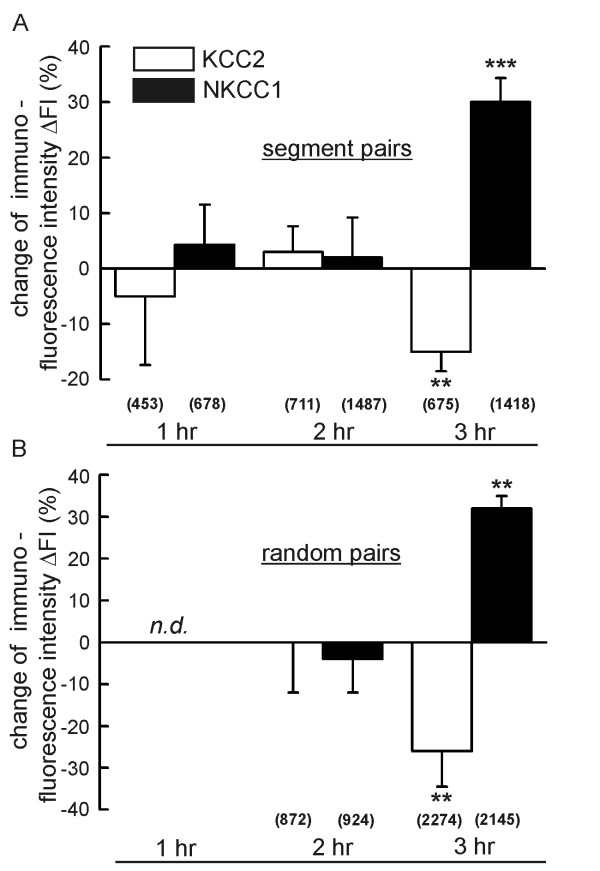
**Altered expression of Cl^- ^transport proteins during treatment with inflammatory mediators**. (A) Results from the segment-pair protocol where the mean immunofluorescence from each test DRG (FI_test_) is related to that of its contralateral control DRG (FI_ctr_). The change of fluorescence intensity ΔFI = (FI_test_-FI_ctr_)/FI_ctr_·100 [%] is depicted 1 hr, 2 hr, and 3 hr of treatment, respectively. The data show that 3 hr after the start of the treatment the expression of the Na^+^, K^+^, 2Cl^- ^cotransporter NKCC1 is enhanced while that of the K^+^, Cl^- ^cotransporter KCC2 is reduced. (B) A similar result was obtained in experiments where control DRGs and test DRGs were not obtained from the same vertebral segment, but were randomly combined. Indicated significance levels are p ≤ 0.01 (**) and p ≤ 0.001 (***). Numbers in parentheses indicate cells evaluated.

We tested for possible effects of the inflammatory mediators on the transcription of NKCC1 and KCC2 by semi-quantitative PCR analysis of the respective mRNAs. Over the 3-hr time course of the experiments, we did not detect any change in mRNA levels of either Cl^- ^transporter *(data not shown)*. This observation suggests that the enhanced Cl^- ^accumulation is not the consequence of transcriptional upregulation of NKCC1 or the downregulation of KCC2 transcription. To study effects of the treatment with inflammatory mediators on the protein level, we performed quantitative immunohistochemistry on 18 T7-T9 DRGs (9 control, 9 treated) from 3 animals. We stained cryosections with antibodies raised against NKCC1 and KCC2, and we compared the mean fluorescence intensity of the stained neurons in each test DRG (FI_test_) to that of the corresponding contralateral control DRG (FI_ctr_). Care was taken to include all intact cell sections with fluorescence above background, so as not to introduce any bias into the evaluation procedure. The results are give as percent change of fluorescence intensity, ΔFI = (FI_test_-FI_ctr_)/FI_ctr_·100 [%]. ΔFI (± SD; 3 animals) was 4.3 ± 7.2% (1 hr), 2 ± 7.2% (2 hr), and 30 ± 4.35% (3 hr) for NKCC1, and -5 ± 12.4% (1 hr), 7 ± 4.6% (2 hr), and -15 ± 3.5% (3 hr) for KCC2 (Fig. 3A). These data demonstrate that NKCC1 and KCC2 expression levels remain unchanged over 2 hr of treatment with inflammatory mediators. After 3 hr, however, the expression level of KCC2 dropped significantly while that of NKCC1 increased. While the changes in NKCC1 and KCC2 expression are consistent with increased Cl^- ^accumulation, the time course of the expression changes lags behind the rise in [Cl^-^]_i_. Cl^- ^accumulation increases already after 2 hr (Fig. 2B) when the co-transporters are still at control level. To exclude any bias that might originate from our "segment pairs" analysis, we repeated the measurements and compared 6 control DRGs to 6 treated DRGs from different vertebral segments. In these experiments, ΔFI (± SD; 3 animals) was -4 ± 8% (2 hr) and 32 ± 3% (3 hr) for NKCC1, and 0 ± 12% (2 hr) and -26 ± 8,5% (3 hr) for KCC2 (Fig. 3B). Thus, the "random pairs" protocol yielded the same result, with significant effects on the co-transporter expression only after 3 hr. These results demonstrate that inflammatory mediators induce a change in the regime of Cl^- ^handling in DRG neurons with a delay of 3 hr after the start of the inflammatory treatment. The effect of the mediators is consistent with increased Cl^- ^accumulation, as NKCC1 expression is enhanced, while KCC2 expression is attenuated.

### The early rise of intracellular Cl^- ^levels coincides with phosphorylation of NKCC1

**Figure 4:**
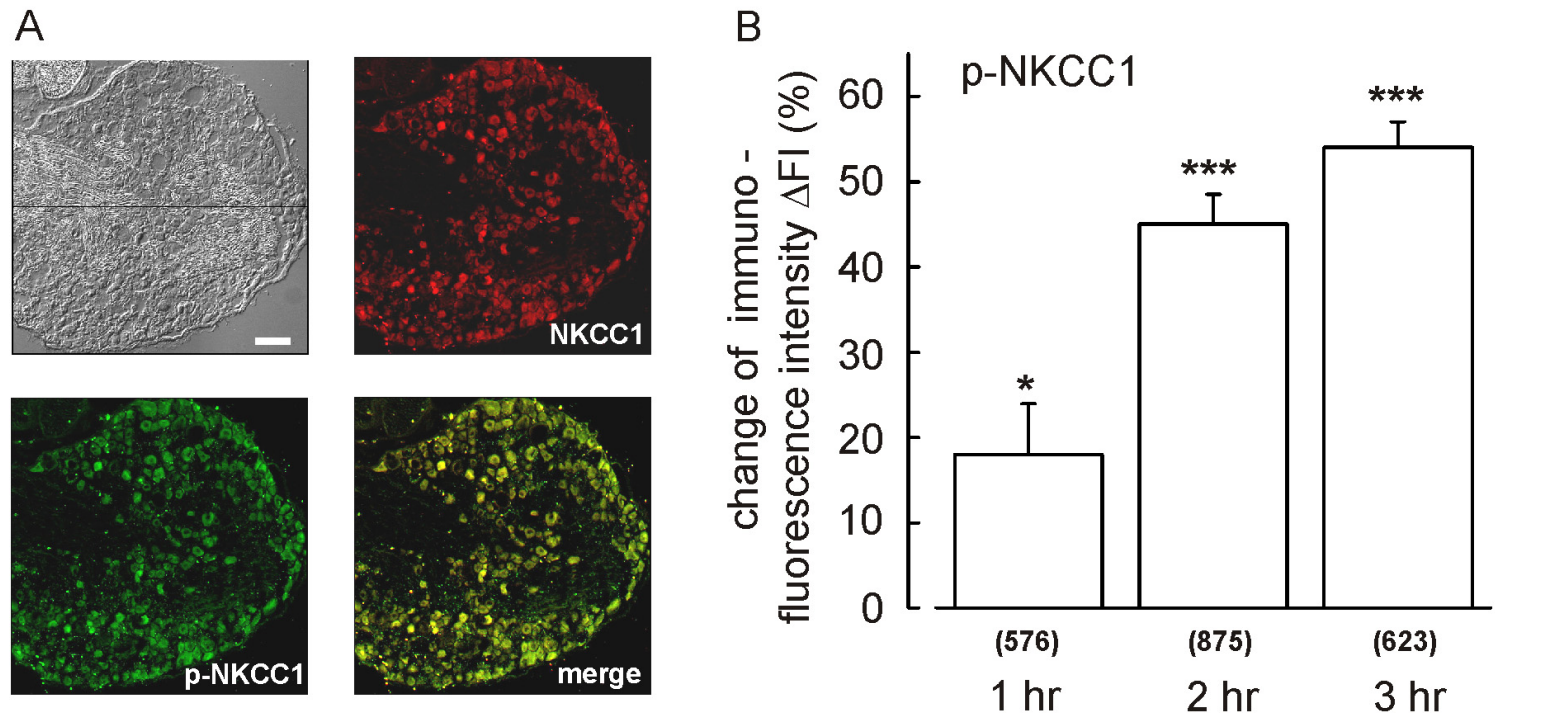
Immunohistochemical analysis of NKCC1 phosphorylation. (A) A cryosection of a T9 DRG was co-stained with antisera raised against NKCC1 and p-NKCC1 after a 3-hr treatment with inflammatory mediators. The two antibodies showed an almost perfect co-staining. The calibration bar indicates 100 μm. (B) In contrast to the NKCC1 immunosignal, the p-NKCC1 signal increased already 1 hr after the start of the treatment. The change of p-NKCC1 immune fluorescence in treated DRGs (FI_test_) compared to the respective contralateral control DRGs (FI_ctr_) is expressed as ΔFI = (FI_test_-FI_ctr_)/FI_ctr_·100 [%]. ΔFI (± SD; 3 animals) was 18 ± 5.9% (1 hr), 45 ± 3.5% (2 hr), and 54 ± 3.3% (3 hr). Indicated significance levels are p ≤ 0.05 (*) and p ≤ 0.001 (***). Numbers in parentheses indicate cells evaluated.

The activity of NKCC1 and KCC2 can be modulated by phosphorylation [26,27]. To test whether NKCC1 phosphorylation may contribute to the early rise of Cl^- ^levels (< 3 hr), we stained 9 DRG sections from 3 animals with an antibody raised specifically against the phosphorylated form of NKCC1 (p-NKCC1). Preadsorption with a peptide corresponding to the non-phosphorylated epitope ensured the specificity of that immunosignal. The antisera directed against NKCC1 (C-14 antibody) and p-NKCC1 showed a virtually perfect match in the DRG sections (Fig. 4A) indicating that both non-phosphorylated and phosphorylated forms of NKCC1 are present in most neurons. However, in contrast to the NKCC1 immunosignal, the p-NKCC1 signal responded rapidly and robustly to the inflammatory mediators. Already after 1 hr, a significant rise of the p-NKCC1 signal (+18% ± 5.9%; p ≤ 0.05) was observed (Fig. 4B). Two hours after addition of the inflammatory mediators, the p-NKCC1 signal was 45% (± 3.5%; p ≤ 0.001) above control, while the total expression level of NKCC1 showed no response (Fig. 3). After 3 hours, the p-NKCC1 signal was increased by 54% (± 3.3%; p ≤ 0.001) relative to control DRGs. These data demonstrate that the inflammatory mediators induce the phosphorylation of NKCC1 in DRG neurons and suggest that this phosphorylation causes the early (< 3 hr) phase of enhanced Cl^- ^accumulation observed by 2P-FLIM in response to the stimulus.

## Discussion

The goal of this study was to establish an *in vitro *assay that monitors effects of inflammatory agents on chloride regulation in DRG neurons. Since we are particularly interested in rapid changes of chloride accumulation and the underlying dynamics of chloride transport proteins, we developed a 3-hr protocol to obtain relevant data. We have treated freshly dissected rat DRGs with a mixture of inflammatory mediators representing the kinins (bradykinin), the prostanoids (PGE_2_), the growth factors (NGF), as well as ATP as a compound released from damaged tissue, to create a medium similar to a site of inflammation. The DRG neurons responded to this treatment with increased expression of SP and CGRP, an effect characteristic for the inflammatory response of peptidergic nociceptors. For example, NGF can induce the expression of SP and CGRP through activation of TrkA receptors, a major pathway of neurotrophin-induced hyperalgesia [42]. DRG neurons showed good viability during the 3-hr experiments. The use of contralateral DRGs as controls in our "segment pair" protocol provided a suitable reference for the statistical evaluation of the experiments.

### Inflammatory mediators stimulate Cl^- ^accumulation in DRGneurons

In a previous paper we have shown that [Cl^-^]_i _in mouse DRG neurons shows a general decrease within the first three postnatal weeks, as Cl^- ^homeostasis undergoes a maturation process [39]. The result of this process is a heterogeneous pattern of Cl^- ^levels among the neurons in each DRG. In the present paper we report that the intracellular Cl^- ^levels are not static but that they can be raised by inflammatory mediators. Our 2P-FLIM data indicate that the mediators induce a uniform rise of intracellular Cl^- ^in most DRG neurons. We have not attempted to obtain a quantitative calibration of absolute Cl^- ^concentrations. Currently available calibration procedures rely on the Cl^-^/OH^- ^exchanger tributyltin [3,39-41]. This compound can be used in isolated cells to dissipate Cl^- ^gradients across the plasma membrane and to set intracellular Cl^- ^to known values. We have previously transferred the 2P-FLIM calibration obtained from isolated DRG neurons to the evaluation of data recorded from intact DRGs [39]. This procedure yielded a broad distribution of [Cl^-^]_i _values, ranging from 20 – 100 mM in adult animals. A comparison of similar 2P-FLIM measurements with X-ray microprobe analysis of [Cl^-^]_i _in olfactory sensory neurons [41] suggested that the two sets of results could differ by ± 20 mM. For the present study we, therefore, attempted to obtain a more direct calibration and used tributyltin in intact DRGs. However, this did not lead to a satisfactory protocol. Treatment with tributyltin did not produce uniform Cl^- ^levels even in the superficial cell layer of the ganglia. Instead, the substance produced prolonged increases of the intracellular Ca^2+ ^concentration which may compromise the validity of the calibration. We suppose that the satellite glial cells which wrap each neuron inside the DRGs [43] prevent the calibration. However, in the present study, we wanted to test the response of DRG neurons to inflammatory mediators while leaving the cellular context as intact as possible – including the satellite glial cells. Our 2P-FLIM results are, therefore, qualitative. They clearly demonstrate an increase of Cl^- ^accumulation in reponse to inflammatory mediators, but they do not give information about which level of [Cl^-^]_i _is reached during the experiments.

The rise of intracellular Cl^- ^during treatment with inflammatory mediators points to a similiar shift in Cl^- ^regulation as observed in various injured neurons [19]. Thus, cortical neurons [44,45] and hippocampal neurons [46,47] show increased Cl^- ^levels and loss of GABA-ergic inhibition in connection with epilepsy. Moreover, nociceptive lamina-I dorsal-horn neurons in a model of neuropathic pain display a depolarized E_Cl _due to increased Cl^- ^accumulation [31] and the consequent occurrence of excitatory Cl^- ^currents. A number of similar observations have led to the concept that various forms of neurological disorders are associated with increased Cl^- ^accumulation and with the resulting failure of GABAergic or glycinergic inhibition [8]. In line with this notion, our 2P-FLIM measurements suggest that inflammation boosts the excitatory potential of Cl^- ^currents in DRG neurons through an increased efficiency of Cl^- ^accumulation.

### Differential effects of inflammatory mediators on Cl^-^transporters

[Cl^-^]_i _levels in neurons essentially reflect the balance of Cl^- ^uptake by NKCC1 and Cl^- ^extrusion by KCC2. While the expression of NKCC1 in DRG neurons is well documented (*e.g*. [13] and references therein), there are conflicting results about the expression of KCC2. Several authors have failed to detect KCC2-mRNA in DRGs by PCR or *in-situ *hybridization [24,30] while others have documented both the KCC2 message and protein in DRGs [23,39]. We consistently found a KCC2 PCR signal, which was weaker than the NKCC1 signal, as well as a reproducible KCC2 immune fluorescence which changed in opposite direction to the NKCC1 signals upon treatment with inflammatory mediators. We therefore assume that the two transporters co-determine [Cl^-^]_i _both at low levels in control DRGs and at high levels in treated DRGs. The temporal correlation between the p-NKCC1 signal and the rise of [Cl^-^]_i _suggests that Cl^- ^uptake by NKCC1 is enhanced within 1–2 hours after treatment with inflammatory mediators through phosphorylation of the Cl^- ^transporter. This rapid response may cause the rise of [Cl^-^]_i _observed by 2P-FLIM two hours after the start of the treatment. On a longer time scale (3 hr) the amount of transporter proteins detectable in DRG neurons changed significantly, as the NKCC1 immunosignal increased and the KCC2 signal decreased. Interestingly, no changes in mRNA levels were observed for either of the two transporters. These data suggest that the inflammatory mediators cause posttranslational changes such as phosphorylation and recruitment of Cl^- ^transporters. Changes of gene transcription do not appear to contribute to the altered Cl^- ^regulation within the time frame of our experiments. This finding is in good accordance with recent results from nociceptive neurons of the dorsal horn. Changes of Cl^- ^regulation that led to disinhibition of these spinal neurons and to referred mechanical hyperalgesia involved a rapid and transient increase of NKCC1 phosphorylation followed by increased recruitment of NKCC1 protein to the plasma membrane within 3 hours [33]. In contrast, the KCC2 expression level in dorsal horn neurons declines upon peripheral inflammation caused by complete Freund's adjuvant injection [35]. KCC2 shows a very high turn-over rate at the plasma membrane (50% of labeled KCC2 is removed from the plasma membrane within 20 min in hippocampal neurons [48]) and, therefore, represents a suitable target for processes that regulate the rate of Cl^- ^extrusion from the cell. Thus, the DRG neurons in our assay recapitulate the disinhibition process of dorsal-horn neurons by increasing Cl^- ^uptake and by attenuating Cl^- ^extrusion.

### Chloride accumulation and inflammatory hyperalgesia

In contrast to most neurons of the central nervous system, the primary afferent neurons of the somatosensory and the olfactory systems accumulate intracellular Cl^- ^and, hence, provide the basis for excitatory Cl^- ^currents. Both types of sensory neurons employ NKCC1 as major Cl^- ^uptake pathway [13,22,49], but experiments with NKCC1^-/- ^knock-out mice revealed that other Cl^- ^transporters, possibly including Cl^-^/HCO_3_^- ^exchangers, contribute as well [13,50-52]. While the somatosensory and olfactory sensory neurons share the propensity to maintain elevated intracellular Cl^- ^levels, they differ in their specific regime of Cl^- ^handling. The olfactory sensory neurons show uniform and efficient Cl^- ^accumulation and maintain their E_Cl _near 0 mV [41,53]. Cl^- ^ions are, consequently, the main charge carriers of the olfactory receptor current [51,54,55], and Cl^- ^accumulation plays a dominant role in the olfactory sensory response. In contrast, the efficiency of Cl^- ^accumulation in DRG neurons appears to be regulated. The E_Cl _across the axonal membrane of DRG neurons is more negative than in olfactory neurons. Taking into account previously measured intracellular Cl^- ^concentrations of DRG neurons in intact ganglia (20 – 100 mM [39]) or in primary culture (20 – 70 mM; [1-3,40,56]), E_Cl _is expected to range between -50 and -10 mV. Importantly, this dynamic range of E_Cl _comprises values where Cl^- ^currents are not – or only mildly – excitatory (-50 mV) and values with strong excitatory power (-10 mV). A shift of E_Cl _during inflammation can, therefore, effectively control the electrical response of DRG neurons.

Our data indicate that the effect of inflammatory mediators on [Cl^-^]_i _is not restricted to nociceptors, as DRG neurons of all sizes respond. In which way could a general increase of [Cl^-^]_i _in DRG neurons contribute to inflammatory hyperalgesia? Both spinal and peripheral processes are likely. The spinal afferents of nociceptors express GABA_A _receptors which mediate PAD by conducting Cl^- ^efflux. When GABA is released from spinal interneurons, a moderate depolarization occurs under normal conditions and causes presynaptic inhibition of the nociceptor. In primary afferent neurons originating from inflamed tissue, the increased [Cl^-^]_i _levels may alter the response to GABA. As a consequence of the increased driving force for Cl^- ^efflux, presynaptic depolarization is thought to be faster and to promote excitation [4,5]. Thus, GABAergic interneurons may facilitate spike generation and, hence, synaptic transmission for afferent signals. Moreover, the GABAergic interneurons could mediate lateral sensitization between adjacent nociceptors, or between nociceptors and low-threshold mechanoreceptors which also increase their [Cl^-^]_i_, giving rise to secondary hyperalgesia or allodynia, respectively. In any case, increasing presynatic [Cl^-^]_i _during inflammation is expected to strengthen afferent sensory signals in DRG neurons and to support hyperalgesia.

Little is known about the role of Cl^- ^channels and Cl^- ^currents in the peripheral sensory endings of DRG neurons. Peripheral GABA can either inhibit or excite nociceptors, depending on the GABA concentration. Cutaneous afferent fibres respond to peripheral GABA application with depolarization in various preparations. Ault and Hildebrand [9] developed an isolated rat spinal cord – tail preparation and used ventral root reflex recordings to test peripheral GABA effects. They found that application of 300 μM GABA to the tail skin evoked similar excitatory responses as 3 μM capsaicin. The morphine-sensitivity of this effect demonstrated that the GABA response was mediated by nociceptors. Carlton and co-workes [10] demonstrated the algogenic effect of peripheral GABA. These authors found expression of the GABA_A _receptor subunits α1 and β2/β3 in a subpopulation (10–14%) of unmyelinated sensory fibers in cat skin. Moreover, injection of the GABA_A _agonist muscimol into the skin attenuated formalin-induced nociceptive behaviors at low concentrations, but enhanced such behaviors at higher concentrations. Furthermore, a study of peripheral analgesic effects of neuroactive steroids provided additional evidence that GABA_A _receptors can contribute to peripheral antinociception [57]. While the precise role of peripheral GABA in the generation or modulation of nociceptive signals remains to be explored, these findings point to a bimodal function of GABA-induced Cl^- ^currents in the periphery. Small Cl^- ^currents tend to attenuate nociception, possibly by promoting the inactivation of voltage-gated Na^+ ^channels. Larger Cl^- ^currents, however, can be excitatory and can elicit nociceptive behavior. In this context, an upregulation of peripheral [Cl^-^]_i _during inflammation is expected to potentiate excitatory GABAergic potentials and to amplify the algogenic effect of peripheral GABA. Thus, both spinal and peripheral changes in Cl^- ^regulation appear to contribute to the modulation of sensory responses in DRG neurons. Concepts for a role of excitatory Cl^- ^currents in nociception may be useful for further studies of inflammatory hyperalgesia.

## Conclusion

We have used an *in vitro *assay to examine effects of inflammatory mediators on chloride homeostasis in rat DRG neurons. We found that the intracellular Cl^- ^concentration rises within 2 hours of treatment. Immunohistochemical experiments revealed that phosphorylation of the Na^+^-K^+^-2Cl^- ^cotransporter NKCC1 coincides with this increase of Cl^- ^levels. After 3 hours of treatment, the efficiency of Cl^- ^accumulation is further enhanced through upregulation of NKCC1 expression and reduced expression of the K^+^-Cl^- ^co-transporter KCC2. Our data demonstrate an impact of inflammatory stimuli on Cl^- ^regulation in DRG neurons and support concepts for the role of Cl^- ^accumulation in the generation of hyperalgesia.

## Methods

### Tissue preparation and *in vitro *assay

Adult rats were anesthetized and killed by cervical transsection. All experimental procedures were performed in accordance with the Animal Protection Law and the guidelines and permissions of Heidelberg University. The spinal column was prepared from its dorsal aspect. After exposing the spinal cord with attached dorsal root ganglia (DRG) and spinal nerves, thoracic and lumbal DRGs were carefully excised and transferred into ice-cold extracellular solution (ES solution: 140 mM NaCl, 5 mM KCl, 1 mM MgCl_2_, 2.5 mM CaCl_2_, 10 mM glucose, 10 mM HEPES, pH 7.4). Only DRGs with intact *dura mater *and residual segments of spinal nerves and nerve roots were used for the subsequent experiments. For the *in vitro *inflammation assay, DRGs were transferred to M10 medium, consisting of Minimal Essential Medium (Sigma M2279) with 10% fetal calf serum (Sigma, F7524), 1% glutamine-penicillin-streptomycin (Sigma, G6784), and 1% non-essential amino acids (Sigma, M7145). This medium was complemented with inflammatory mediators (100 ng/ml NGF, 1.8 μM ATP, 0.9 μM bradykinin, and 1,4 μM PGE_2_), and DRGs were treated at 37°C, 5% CO_2_, for 1 – 3 hours. Control DRGs were incubated in M10 medium without the inflammatory mediators.

### Vitality tests

Following the treatment or control periods, DRGs were immobilized in 4% low-melting point agarose in aerated ES solution on ice. Slices were prepared using a vibratome (150 – 170 μm thick) which were then incubated either with 1.5 μM propidium iodide in ES solution (Sigma P4170; 1–2 min, room temperature) or in ES solution containing 10 μM C_12_-resazurin (Invitrogen L34951 plus 1 mM probenicide; 10 – 15 min, 37°C). To stop the reaction, sections were transferred to ES solution. Propidium iodide can only enter dead or damaged cells where it intercalates in the DNA. This causes a shift of the emission maximum from 590 nm to 617 nm and a 20 to 30-fold increase of fluorescence. C_12_-resazurin is reduced to the red fluorescent C_12_- resorufin by NADH only in living cells.

### Two-photon fluorescence-lifetime imaging microscopy(2P-FLIM)

DRGs were loaded with the Cl^- ^indicator dye MQAE (*N*-6-methoxyquinolinium acetoethylester; Molecular Probes, Invitrogen). The dye was dissolved at 5 mM in M10 medium, and DRGs were incubated for 1 hour (the 2^nd ^or 3^rd ^hour of treatment with inflammatory mediators). DRGs were then immobilized in 4% low-melting point agarose in aerated bicarbonate solution (120 mM NaCl, 2.5 mM KCl, 1.25 mM NaH_2_PO_4_, 25 mM NaHCO_3_, 1 mM MgCl_2_, 2 mM CaCl_2_, pH 7.4, aerated with 95% O_2_/5% CO_2_), and observed on the 2P-FLIM instrument at room temperature. MQAE molecules reach the excited state upon absorption of a single ultraviolet photon (λ = 375 nm) or, alternatively, the simultaneous absorption of two infrared photons (λ = 750 nm). We used two-photon excitation to achieve an optical resolution of ~0.5 μm (x/y plane) and 1 μm (z axis). The infrared light used for two-photon excitation caused no detectable photodamage, even with the relatively long observation times of multiple images with 1 min of illumination per image. For the MQAE molecule, the dwell time in the excited singlet state (the fluorescence lifetime, τ) is near 30 ns in water containing 50 μM MQAE and is reduced by anions through collisional quenching. The Cl- dependence of τ is described by the Stern-Volmer relation (τ_0_/τ = 1 + KSV [Cl-]i), where τ_0 _is the fluorescence lifetime in 0 mM Cl-, and KSV, the Stern-Volmer constant, is a measure of the Cl- sensitivity of MQAE. KSV has a value of 185 M-1 in water but only 3 M-1 inside isolated DRG neurons [40]. This reduced sensitivity of intracellular MQAE may result in part from interactions of the dye with other soluble anions and from self-quenching of MQAE at concentrations > 100 μM [40]. In the present study, we did not determine KSV for the neurons in intact DRGs. We, therefore, obtained only information about changes in intracellular Cl-, but do not determine the absolute Cl- concentration. The DRGs were placed on the stage of an upright fluorescence microscope (BX50WI; Olympus Optical, Japan) and observed through a 60× water-immersion objective (n.a. = 0.9; Olympus Optical). Fluorescence was excited with 150 fs light pulses (λ = 750 nm) applied at sufficient intensity to generate two-photon excitation. Light pulses were generated at a frequency of 75 MHz by a mode-locked Titan-Sapphire laser (Mira 900; output power, > 500 mW; Coherent, Santa Clara, USA), which was pumped by the frequency-doubled output (532 nm) of a Nd-vanadate laser (Verdi; Coherent). The laser light was directed through the objective onto the DRGs at reduced power (2.5 mW) using a beam scanner (TILL Photonics, Munich, Germany). Fluorescence was recorded by photomultipliers, and lifetime analysis was performed using electronics (SPC-730; Becker & Hickl, Berlin, Germany) and software (SPC7.22; Becker & Hickl) for time-correlated single-photon counting. DRGs which were not loaded with MQAE did not produce background signals with this method. Lifetime images were analyzed using SPCImage 1.8 and 2.6 (Becker & Hickl) and a self-made image analysis software. A detailed description of the instrument is given elsewhere [40]. Images were obtained by scanning the excitation light focus through tissue layers 1 – 3 cells below the dura mater. Mean values of Cl- concentrations are given with standard deviations (± SD).

### Immunohistochemistry

Following treatment (or control), DRGs were washed in phosphate-buffered solution (PBS: 8.1 mM Na_2_HPO_4_, 1.9 mM NaH_2_PO_4_, 130 mM NaCl, pH 7.4) and then fixed in PBS containing 4% paraformaldehyde for 30 min. After three washing steps in PBS, the tissue was sequentially dehydrated in 10% to 30% sucrose overnight. The DRGs were then embedded in Tissue-Tek (Leica, Nussloch, Germany) and frozen onto the cryostate stage (Leica CM 3050 S) and stored at -20°C. Cryosections (14 μm) were prepared at -20°C and collected on coated slides (SuperFrost^®^Plus, Menzel, Braunschweig, Germany). Sections were air-dried for 30 min, post-fixed in 4% paraformaldehyde in PBS, washed three times in PBS and blocked in 5% ChemiBLOCKER™ (Millipore, Billerica, USA), 0,5% Triton X100 in PBS. Primary antibodies were diluted in the same buffer and incubated for 2 h. The following antibodies and dilutions were used: goat anti-NKCC1, 1:20 (C-14, Santa Cruz Biotechnology, Heidelberg, #sc-21547), goat anti-NKCC1, 1:20 (N-16; Santa Cruz, #sc-21545), goat anti-KCC2, 1:20 (R-14, Santa Cruz, #sc-19420), sheep anti-p-NKCC1, 1:20 (directed against the phosphorylated sites Thr203+Thr207+Thr212 of human NKCC1, The University of Dundee, Scotland, UK), rabbit anti-substance P, 1:50 (PC232L, Oncogene Research, San Diego), and rabbit anti-CGRP, 1:50 (PC205L, Oncogene Research). After three washing steps in PBS, sections were incubated for 90 min with fluorescent labeled secondary antibodies: donkey anti-goat Alexa488 (Invitrogen, A-11055, 1:500), donkey anti-goat Alexa568 (Invitrogen, A-11057, 1:500), donkey anti-sheep Alexa488 (Invitrogen, A-11015, 1:500), donkey anti-rabbit Alexa488 (Invitrogen, A-21206, 1:500). After three washing steps in PBS, a 0.3 μM DAPI solution (Fluka, #32670) was used to stain the nuclei. Sections were coverslipped with Aqua Poly/Mount (Polysciences, Warrington, USA) and analyzed using a Nikon Eclipse 90i upright automated microscope equipped with a Nikon DS-1QM CCD camera. The instrument was used at the Nikon Imaging Center at the University of Heidelberg. No fluorescence signal was observed upon omission of primary antibodies. To test for the specificity of primary antibodies, preadsorption controls were performed for the NKCC1 (N-16) and the KCC2 (R-14) polyclonal antibodies using a 5-fold excess of blocking peptides (0.05 μg/μl) according to the manufacturers specifications. The specificity of the antibodies for NKCC1 (C-14) and p-NKCC1 were confirmed by co-staining with NKCC1 (N-16).

### Semi-quantitative RT-PCR

Total RNA was extracted from DRGs after various durations of treatment with inflammatory mediators, following an established protocol [58]. After DNase I treatment (RNase-free, Fermentas, StLeon-Rot, Germany) cDNA was synthesized from 5 μg total RNA using an oligo-dT primer and Superscript™ III reverse transcriptase (Invitrogen) according to the manufacturer's instructions. The cDNA was quantified for normalization using PCR (22 to 35 cycles) with β-actin primers. The annealing temperature for all primers was 58°C. Semiquantitative PCR amplification was performed on 0.5 μl single-stranded cDNA product with 2U Taq DNA polymerase (Fermentas). The cycling conditions were 94°C for 3 min, 94°C for 30 s, 58°C for 30 sec, 72°C for 1 min for 28 and 32 cycles, respectively, and 72°C for 8 min. Primer pairs were: NKCC1/F 5'CGA ATT ATT GGA GCC ATT ACA GT3', NKCC1/R 5'ACA TCT GGA AAG CTG GGT AGA TA3', KCC2/F 5'GTC TCT GGG CCC GGA GTT T3', KCC2/R 5'GGC ATC CCG CAG GTC TC3'. For positive controls and normalization β-actin primer pairs were: actin/F 5'GGT CAT CAC TAT CGG CAA TGA GC3', actin/R 5'GGA CAG TGA GGC CAG GAT AGA GC3'. The resulting PCR products were cloned into pGMT vector (Promega, Mannheim, Germany) and sequenced.

## Abbreviations

2P-FLIM: two-photon fluorescence-lifetime microscopy; CGRP: calcitonin-gene related peptide; DRG: dorsal root ganglion; GABA: γ-amino butyric acid; KCC: K^+^, Cl^- ^cotransporter; MQAE: *N*-6-methoxyquinolinium acetoethylester; NKCC: Na^+^, K^+^,2Cl^- ^cotransporter; PAD: primary afferent depolarization; SP: substance P

## Competing interests

The authors declare that they have no competing interests.

## Authors' contributions

KF and AW did the immunohistochemistry and, together with TG, the 2P-FLIM measurements. CFW and FM did the PCR experiments. SF wrote, with the help of all other authors, the manuscript. All authors read and approved the final manuscript.

## References

[B1] Alvarez-Leefmans FJ, Gamino SM, Giraldez F, Nogueron I (1988). Intracellular chloride regulation in amphibian dorsal root ganglion neurons studied with ion-sensitive microelectrodes. J Physiol.

[B2] Kenyon JL (2000). The reversal potential of Ca^2+^-activated Cl^- ^currents indicates that chick sensory neurons accumulate intracellular Cl^-^. Neurosci Lett.

[B3] Rocha-Gonzalez HI, Mao S, Alvarez-Leefmans FJ (2008). Na^+^, K^+^, 2Cl^- ^cotransport and intracellular chloride regulation in rat primary sensory neurons: thermodynamics and kinetic aspects. J Neurophysiol.

[B4] Willis WD (1999). Dorsal root potentials and dorsal root reflexes: a double-edged sword. Exp Brain Res.

[B5] Cervero F, Laird JMA, García-Nicas E (2003). Secondary hyperalgesia and presynaptic inhibition: an update. Eur J Pain.

[B6] Cervero F, Laird JMA (1996). Mechanisms of touch-evoked pain (allodynia): a new model. Pain.

[B7] Rudomin P, Schmidt RF (1999). Presynaptic inhibition in the vertebrate spinal cord revisited. Exp Brain Res.

[B8] De Koninck Y (2007). Altered chloride homeostasis in neurological disorders: a new target. Curr Opin Pharmacol.

[B9] Ault B, Hildebrand LM (1994). GABA_A _receptor-mediated excitation of nociceptive afferents in the rat isolated spinal cord-tail preparation. Neuropharmacology.

[B10] Carlton SM, Zhou S, Coggeshell RE (1999). Peripheral GABA_A _receptors: evidence for peripheral primary afferent depolarization. Neurosci.

[B11] Pieraut S, Laurent-Matha V, Sar C, Hubert T, Mechaly I, Hilaire C, Mersel M, Delpire E, Valmier J, Scamps F (2007). NKCC1 phosphorylation stimulates neurite growth of injured adult sensory neurons. J Neurosci.

[B12] Price TJ, Cervero F, de Koninck Y (2005). Role of cation-chloride-cotransporters (CCC) in pain and hyperalgesia. Curr Top Med Chem.

[B13] Sung KW, Kirby M, McDonald MP, Lovinger DM, Delpire E (2000). Abnormal GABA_A _receptor-mediated currents in dorsal root ganglion neurons isolated from Na-K-2Cl cotransporter null mice. J Neurosci.

[B14] Laird JMA, García-Nicas E, Delpire EJ, Cervero F (2004). Presynaptic inhibition and spinal pain processing in mice: a possible role for the NKCC1 cation-chloride co-transporter in hyperalgesia. Neurosci Lett.

[B15] Pitcher MH, Price TJ, Entrena JM, Cervero F (2007). Spinal NKCC1 blockade inhibits TRPV1-dependent referred allodynia. Mol Pain.

[B16] Willis EF, Clough GF, Church MK (2004). Investigation into the mechanisms by which nedocromil, furosemide and bumetanide inhibit the histamine-induced itch and flare response in human skin *in vivo*. Clin Exp Allergy.

[B17] Granados-Soto V, Arguelles CF, Alvarez-Leefmans FJ (2005). Peripheral and central antinociceptive action of Na^+ ^-K^+ ^-2Cl^- ^cotransporter blockers on formalin-induced nociception in rats. Pain.

[B18] Valencia-de Ita S, Lawand NB, Lin Q, Castaneda-Hernandez G, Willis WD (2006). Role of the Na^+ ^-K^+ ^-2Cl^- ^contransporter in the development of capsaicin-induced neurogenic inflammation. J Neurophysiol.

[B19] Payne JA, Rivera C, Voipio J, Kaila K (2003). Cation-chloride co-transporters in neuronal communication, development and trauma. Trends Neurosci.

[B20] Gamba G (2005). Molecular physiology and pathophysiology of electroneutral cation-chloride cotransporters. Physiol Rev.

[B21] Rivera C, Voipio J, Kaila K (2005). Two developmental switches in GABAergic signalling: the K^+^-Cl^- ^cotransporter KCC2 and carbonic anhydrase CAVII. J Physiol.

[B22] Alvarez-Leefmans FJ, León-Olea M, Mendoza-Sotelo J, Alvarez FJ, Anton B, Garduno R (2001). Immunolocalization of the Na^+^-K^+^-2Cl^- ^cotransporter in peripheral nervous tissue of vertebrates. Neuroscience.

[B23] Lu J, Karadsheh M, Delpire E (1999). Develeopmental regulation of the neuronal-specific isoform of K-Cl cotransporter KCC2 in postnatal rat brains. J Neurobiol.

[B24] Kanaka C, Ohno K, Okabe A, Kuriyama K, Itoh T, Fukuda A, Sato K (2001). The differential expression patterns of messenger RNAs encoding K-Cl cotransporters (KCC1,2) and Na-K-2Cl cotransporter (NKCC1) in the rat nervous system. Neuroscience.

[B25] Schomberg SL, Bauer J, Kintner DB, Su G, Flemmer A, Forbush B, Sun D (2003). Cross talk between GABA_A _receptor and the Na-K-Cl cotransporter is mediated by intracellular Cl^-^. J Neurophysiol.

[B26] Gagnon KBE, England R, Delpire E (2006). Characterization of SPAK and OSR1, regulatory kinases of the Na-K-2Cl cotransporter. Mol Cell Biol.

[B27] Giménez I (2006). Molecular mechanisms and regulation of furosemide-sensitive Na-K-Cl cotransporters. Curr Opin Nephrol Hypertens.

[B28] Blaesse P, Guillemin I, Schindler J, Schweizer M, Delpire E, Khirough L, Friauf E, Nothwang HG (2006). Oligorimerization of KCC2 correlates with development of inhibitory neurotransmission. J Neurosci.

[B29] Prescott SA, Sejnowski TJ, De Koninck Y (2006). Reduction of anion reversal potential subverts the inhibitory control of firing rate in spinal lamina I neurons: towards a biophysical basis for neuropathic pain. Mol Pain.

[B30] Morales-Aza BM, Chillingworth NL, Payne JA, Donaldson LF (2004). Inflammation alters cation chloride cotransporter expression in sensory neurons. Neurobiol Dis.

[B31] Coull JAM, Boudreau D, Bachand K, Prescott SA, Nault F, Sik A, De Koninck P, De Koninck Y (2003). Trans-synaptic shift in anion gradient in spinal lamina I neurons as a mechanism of neuropathic pain. Nature.

[B32] Coull JAM, Beggs S, Boudreau D, Boivin D, Tsuda M, Inoue K, Gravel C, Salter MW, De Koninck Y (2005). BDNF from microglia causes the shift in neuronal anion gradient underlying neuropathic pain. Nature.

[B33] Galan A, Cervero F (2005). Painful stimuli induce *in vivo *phosphorylation and membrane mobilization of mouse spinal cord NKCC1 co-transporter. Neuroscience.

[B34] Nomura H, Sakai A, Nagano M, Umino M, Suzuki H (2006). Expression changes of cation chloride cotransporters in the rat spinal cord following intraplantar formalin. Neurosci Res.

[B35] Zhang W, Liu L-Y, Xu T-L (2008). Reduced potassium-chloride co-transporter expression in spinal cord dorsal horn neurons contributes to inflammatory pain hypersensitivity in rats. Neuroscience.

[B36] Bulling DG, Kelly D, Bond S, McQueen DS, Seckl JR (2001). Adjuvant-induced joint inflammation causes very rapid transcription of beta-preprotachykinin and alpha-CGRP genes in innervating sensory ganglia. J Neurochem.

[B37] Callsen-Cencic P, Mense S (1997). Expression of neuropeptides and nitric oxide synthase in neurones innervating the inflamed rat urinary bladder. J Auton Nerv Syst.

[B38] Xu P, Van Slambrouck C, Berti-Mattera L, Hall AK (2005). Activin induces tactile allodynia and increases calcitonin gene-related peptide after inflammation. J Neurosci.

[B39] Gilbert D, Franjic-Würtz C, Funk K, Gensch T, Frings S, Möhrlen F (2007). Differential maturation of chloride homeostasis in primary afferent neurons of the somatosensory system. Int J Dev Neurosci.

[B40] Kaneko H, Putzier I, Frings S, Gensch T, Fuller CM (2002). Determination of intracellular chloride concentration in dorsal root ganglion neurons by fluorescence lifetime imaging. Calcium-activated chloride channels.

[B41] Kaneko H, Putzier I, Frings S, Kaupp UB, Gensch T (2004). Chloride accumulation in mammalian olfactory sensory neurons. J Neurosci.

[B42] Shu X-Q, Mendell LM (1999). Neurotrophins and hyperalgesia. Proc Nat Acad Sci USA.

[B43] Hanani M (2005). Satellite glial cells in sensory ganglia: from form to function. Brain Res Brain Res Rev.

[B44] Cohen I, Navarro V, Clemenceau S, Baulac M, Miles R (2002). On the origin of inerictal activity in human temporal lobe epilepsy *in vitro*. Science.

[B45] Jin X, Huguenard JR, Prince DA (2005). Impaired Cl^- ^extrusion in layer V pyramidal neurons of chronically injured epileptogenic neocortex. J Neurophysiol.

[B46] Palma E, Amici M, Sobrero F, Spinelli G, Di AS, Ragozzino D, Mascia A, Scoppetta C, Esposito V, Miledi R, Eusebi F (2006). Anomalous levels of Cl^- ^transporters in the hippocampal subiculum from temporal lobe epilepsy patients make GABA excitatory. Proc Natl Acad Sci U S A.

[B47] Rivera C, Li H, Thomas-Crusells J, Lahtinen H, Viitanen T, Nanobashvili A, Kokaia Z, Airaksinen MS, Voipio J, Kaila K, Saamba M (2002). BDNF-induced TrkB activation down-regulates the K^+^-Cl^- ^cotransporter KCC2 and impairs neuronal Cl^- ^extrusion. J Cell Biol.

[B48] Rivera C, Viopio J, Thomas-Crusells J, Li H, Emri Z, Sipilä S, Payne JA, Minichiello L, Saarma M, Kaila K (2004). Mechanism of activity-dependent downregulation of the neuron-specific K-Cl cotransporter KCC2. J Neurosci.

[B49] Reisert J, Lai J, Yau K-W, Bradley J (2005). Mechanism of excitatory Cl^- ^response in mouse olfactory receptor neurons. Neuron.

[B50] Nickell WT, Kleene NK, Gesteland RC, Kleene SJ (2006). Neuronal chloride accumulation in olfactory epithelium of mice lacking NKCC1. J Neurophysiol.

[B51] Nickell WT, Kleene NK, Kleene SJ (2007). Mechanisms of neuronal chloride accumulation in intact mouse olfactory epithelium. J Physiol.

[B52] Smith DW, Thach S, Marshall EL, Mendoza MG, Kleene SJ (2008). Mice lacking NKCC1 have normal olfactory sensitivity. Physiol Behav.

[B53] Reuter D, Zierold K, Schröder W, Frings S (1998). A depolarizing chloride current contributes to chemo-electrical transduction in olfactory sensory neurons *in situ*. J Neurosci.

[B54] Reisert J, Bauer PJ, Yau K-W, Frings S (2003). The Ca-activated Cl channel and its control in rat olfactory receptor neurons. J Gen Physiol.

[B55] Boccaccio A, Menini A (2007). Temporal development of cyclic nucleotide-gated and Ca^2+^-activated Cl^- ^currents in isolated mouse olfactory sensory neurons. J Neurophysiol.

[B56] Gallagher JP, Higashi H, Nishi S (1978). Characterization and ionic basis of GABA-induced depolarizations recorded in vitro from cat primary afferent neurones. J Physiol Lond.

[B57] Pathirathna S, Brimelow BC, Jagodic MM, Krishnan K, Jiang X, Zorumski CF, Mennerick S, Covey DF, Todorovic SM, Jevtovic-Todorovic V (2005). New evidence that both T-type calcium channels and GABA_A _channels are responsible for the potent peripheral analgesic effects of 5α-reduced neuroactive steroids. Pain.

[B58] Chomczynski P, Sacchi N (1987). Single-step method of RNA isolation by acid guanidinium thiocyanate-phenol-chloroform extraction. Anal Biochem.

